# Congenital Anatomical Variant with Cranial Origin of Internal Iliac Arteries

**DOI:** 10.1055/s-0041-1725089

**Published:** 2021-10-07

**Authors:** Umberto G. Rossi, Anna M. Ierardi, Maurizio Cariati

**Affiliations:** 1 Department of Diagnostic Imaging, Interventional Radiology Unit, Ente Ospedaliero Galliera Hospital, Genova, Italy; 2 Department of Diagnostic and Therapeutic Advanced Technology, Diagnostic and Interventional Radiology Unit, Azienda Socio Sanitaria Territoriale Santi Paolo and Carlo Hospital, Milan, Italy

**Keywords:** iliac artery, vascular, variant, congenital, anatomy, imaging

## Abstract

We report the case of a 73-year-old male who underwent abdominal multidetector computed tomography with vascular reconstruction that highlighted a congenital variant of iliac arteries. Iliac artery anatomical variants are exceedingly rare and only a few cases have been reported in the literature.

We present a case of a 73-year-old male who presented to our emergency department for abdominal trauma due to a car incident. He underwent abdominal multidetector computed tomography that excluded abdominal traumatic pathologies. Vascular coronal volume rendering reconstruction highlighted an asymptomatic congenital anatomical variant, with cranial origin of internal iliac arteries ( Fig. 1 ). This proximal origin of both internal iliac arteries was associated with a reduced length of both common iliac arteries: right one of 16 mm and left one of 4 mm. The patient showed no traumatic signs, and he was discharged after 6 hours of observation.

**Fig. 1:**
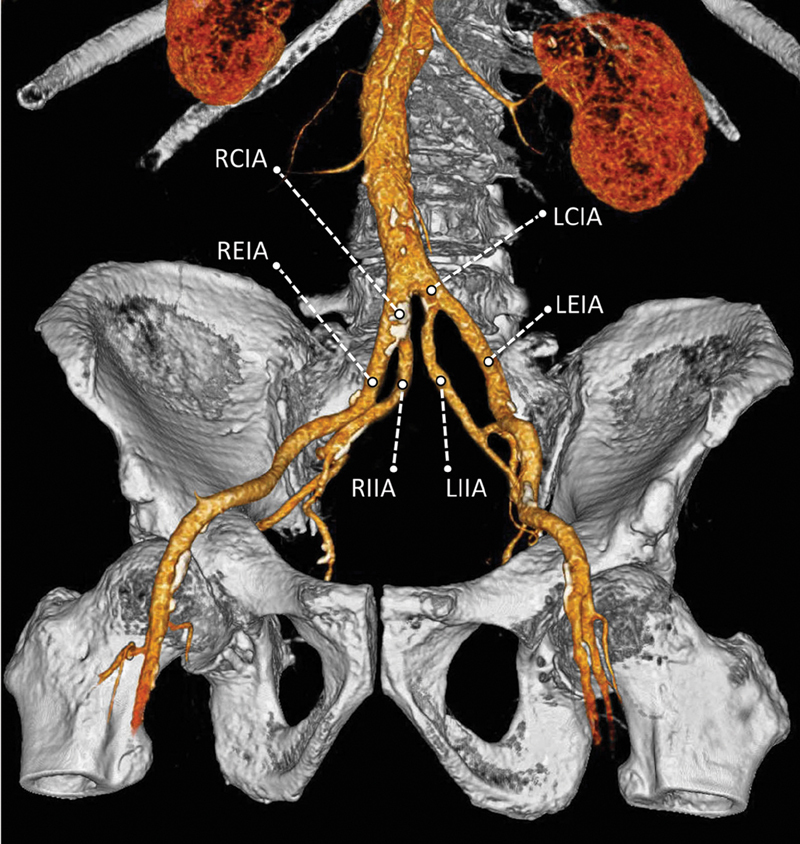
Multidetector computed tomography angiography coronal volume reconstruction demonstrating the presence of a congenital anatomical variant, with cranially displaced origin of the internal iliac arteries (right internal iliac artery [RIIA] and left internal iliac artery [LIIA]), reduced length of both common iliac arteries (right common iliac artery [RCIA] and left common iliac artery [LCIA]), and increased length of both external iliac arteries (right external iliac artery [REIA] and left external iliac artery [LEIA]).

Congenital variants of the iliac arteries are exceedingly rare, and only a few cases have been reported in the literature. 1 2 3 Iliac vasculature variants arise during the embryological process, starting in the fourth week of gestation. 1 These iliac variants can be classified into three categories: 2 (1) group 1 includes variants of origin and/or course; (2) group 2 involves hypoplasia or atresia, with a persistent sciatic artery; and (3) group 3 represents isolated hypoplasia or atresia. Group 1 variants are generally coincidental findings because the subjects are asymptomatic. 1 2 3

With an increase in noninvasive diagnostic vascular imaging, thoracoabdominal vascular anomalies are seen with greater frequency, 3 4 5 6 also in asymptomatic patients. Knowledge of possible variations in iliac vascular anatomy, in terms of origin and course or hypoplasia, is crucial for patients who are candidates for abdominal vascular and endovascular treatments.
